# Effect of Long-Time Annealing at 1000 °C on Phase Constituent and Microhardness of the 20Co-Cr-Fe-Ni Alloys

**DOI:** 10.3390/ma12101700

**Published:** 2019-05-25

**Authors:** Changjun Wu, Ya Sun, Ya Liu, Hao Tu

**Affiliations:** 1 Jiangsu Key Laboratory of Materials Surface Science and Technology, Changzhou University, Jiangsu 213164, China; ya_sun@aliyun.com (Y.S.); yliu@cczu.edu.cn (Y.L.); tuhao@cczu.edu.cn (H.T.); 2 National Experimental Teaching Demonstration Center of Materials Science and Engineering, School of Materials Science and Engineering, Changzhou University, Jiangsu 213164, China; 3 Jiangsu Collaborative Innovation Center of Photovoltaic Science and Engineering, Changzhou University, Jiangsu 213164, China

**Keywords:** Co-Cr-Fe-Ni, high entropy alloys, annealing, phase constituent, microhardness

## Abstract

The phase constituent and microhardness of the arc-melted 20Co-Cr-Fe-Ni alloys, in both as-cast state and after annealing at 1000 °C for 30 days, were experimentally investigated by scanning electron microscopy (SEM) and X-ray diffraction (XRD). Experiment results indicated that a uniform, stable, single Face-Center Cubic (FCC) phase can be obtained in as-cast 20 Co-Cr-Fe-Ni alloys with less than 30 at.% Cr. Annealing at 1000 °C has no effect on their phase composition and microhardness. When the Cr content is above 40 at.%, the σ phase forms and its volume fraction increases with the Cr content, which leads to an increase in microhardness. Annealing at 1000 °C for 30 days can slightly decrease the volume fraction of the σ phase and slightly decrease the alloy microhardness. Except for the Fe-rich alloys, the alloy microhardness increases with the Cr content when the Co and Ni or the Co and Fe contents were fixed. Moreover, comparing with the thermodynamically calculated phase diagram based on the TCFE database, it has been proved that the calculation can predict the phase stability of the FCC phase and the 1000 °C isothermal section. However, it fails to predict the stability of the σ phase near the liquidus. The present results will help to design and process treatment of the Co-Cr-Fe-Ni based high entropy alloys.

## 1. Introduction

Recently, high-entropy alloys (HEAs), or named as multi-component alloys, have been widely concerned [1,2,3]. Among them, CoCrFeNi-based alloys are the most popular [4,5]. Because the equal-atomic CoCrFeNi alloy can keep stable FCC (Face-Center Cubic) single-phase at different annealing temperature [6]. Adding one or two elements into the CoCrFeNi alloy can promote its strength [7,8,9,10,11,12], such as CoCrFeNiMn [5,13,14,15,16], Al_x_CoCrFeNi [17,18], CoCrCuFeNiTi_x_ [19,20], CoCrFeNiTi_0.5_ [19,20], CrFeCoNiCu [21,22]. Of course, the phase constituent, microstructure and mechanical properties also change with the element addition. 

As a basic alloy system, some Co-Cr-Fe-Ni alloys, either equal molar or non-equal molar, were investigated [23,24,25,26]. Non-equal molar HEAs also have a good performance. However, the phase constituent at the center of the Co-Cr-Fe-Ni system has not been well investigated. He et al. [27] calculated the single FCC phase existed in the central region by the CALPHAD (CALculation of PHase Diagrams) method. Fang et al. [28] proved that the non-equal molar Co*_x_*Cr_50−*x*_Fe_25_Ni_25_ (*x* = 20–35 at.%) alloys was single FCC phase and had a better strength-ductility combination when the Co content reached 35 at.%. However, the prediction of the Co-Cr-Fe-Ni alloys was based on the available Fe-based or Ni-based thermodynamic database, and the phase boundary in the central of the system still needs to be checked.

Moreover, HEAs have good application prospects at high temperature. However, there is no information about the long-time annealed Co-Cr-Fe-Ni alloys at high temperature. Long-time annealing at high temperature can uniform the alloy, promote nanoscale precipitation and reduce the defeats [29,30,31,32,33,34], and also can check the phase stabilities. Moreover, heat treatment can also improve mechanical properties of HEAs. The annealing can also separate the oversaturated elements in solid solution and stabilize the phase [35] and create entire recrystallization to promote the growth of grains [36,37]. Wang et al. [38] found that the σ phase formed in the Al_x_CoCrFeNi alloys with FCC plus BCC (Body-Center Cubic) phases after annealing at over 600 °C, which can harden the alloys. Recently, Guo et al. [39] have concluded that cold rolling combining subsequent annealing is a valid method to refine the Al_0.5_CoCrFeNi alloys. 

At present, much works are focused on the as-cast or short time annealed alloys. The phase stability and mechanical property of the alloys after annealing at high temperature for a long time are unclear. Moreover, the phase boundary of the Co-Cr-Fe-Ni system is still not well investigated. Therefore, the phase constituent and microhardness of the Co-Cr-Fe-Ni alloys at both as-cast and annealing states will be investigated in the present work. It will help to design and process treatment of the Co-Cr-Fe-Ni based high entropy alloys.

## 2. Materials and Methods 

To study the effect of composition and long-time annealing on the phase constituent and microhardness of the Co-Cr-Fe-Ni alloys. Three series of Co-Cr-Fe-Ni alloys were designed in the present work, i.e., Co_2_Cr*_x_*Fe_7−*x*_Ni, Co_2_Cr*_y_*Fe_6−*y*_Ni_2_, Co_2_Cr_z_FeNi_7-z_. The Co content was fixed at 20 at.%. All of the metal elements (Cr, Fe, Co, Ni) used for casting have the purities higher than 99.9 wt.%. The designed alloys with a total mass of 10 g were prepared by the arc-melting casting method in an atmosphere of purified argon. It is necessary to repeated melting for at least four times to ensure the chemical homogeneity. The weight loss is less than 0.1% and can be ignored. These specimens in the shape of flying saucers with a diameter of 10 mm and high of 5 mm were then cut into two pieces: one part was analyzed in as-cast state, and another one was sealed in an evacuated quartz tube and then annealed at 1000 °C for 30 days. At the end of the treatment, the alloys were quenched in cold water. Differently from the as-cast alloys, the 1000 °C annealed alloys were noted as A’#.

To conduct the observation of microstructures, the specimen was ground using silicon carbide papers with #400–#2000 grit, and finally, a 1 μm diamond suspension was used to perform surface polishing. The specimens without etching were examined by JSM-6510 scanning electron microscope (SEM, JEOL, Tokyo, Japan) equipped with an Oxford INCA energy dispersive spectrometer (EDS, Oxford Instruments, Abingdon, UK). The structure of the alloys was characterized by X-ray diffraction (XRD) using a D/max 2500 PC X-ray diffractometer (Rigaku, Tokyo, Japan) with Cu K_α_ radiation and a 2*θ* step size of 0.02°. Microhardness measurements were conducted using a Vicker’s hardness tester (HXD-1000TMC/LC, Sanfeng, Shanghai, China) under a load of 1.96 N and stayed for 20 s.

## 3. Results and Discussion

**Figure 1:**
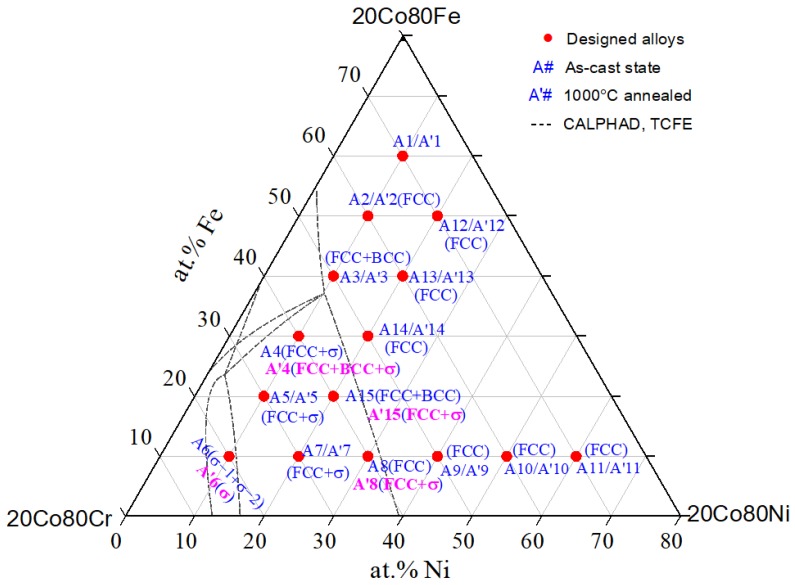
The detected phases in the as-cast and 1000 °C annealed 20Co-Cr-Fe-Ni alloys in the present work. The BCC phase was found in alloy A1 for there is an FCC→BCC transformation when cooling to the room temperature. The dotted line is the calculated phase region boundary of the 20Co-Cr-Fe-Ni system at 1000 °C based on the TCFE thermodynamic database [40].

Firstly, the chemical composition of each alloy was checked by SEM-EDS. The results indicated that the difference between the actual composition and the designed composition is less than 1%, which is within the EDS sensitivity. The detected phases in the as-cast and 1000 °C annealed 20 Co-Cr-Fe-Ni alloys are presented in Figure 1. We divided the alloys into two categories: with single FCC phase and do not contain a single FCC phase.

### 3.1. The Alloys With Single FCC Phase

Except for the alloys A4 (20Co40Cr30Fe10Ni), A8(20Co40Cr10Fe30Ni) and A15 (20Co40Cr20Fe20Ni), the phase constituent of other alloys after 1000 °C annealing is the same as that in as-cast state. To clearly analyze the experiment results, the 1000 °C isothermal section of the 20Co-Cr-Fe-Ni system was calculated based on the TCFE database [40] and is also presented in Figure 1 with dotted lines.

**Figure 2:**
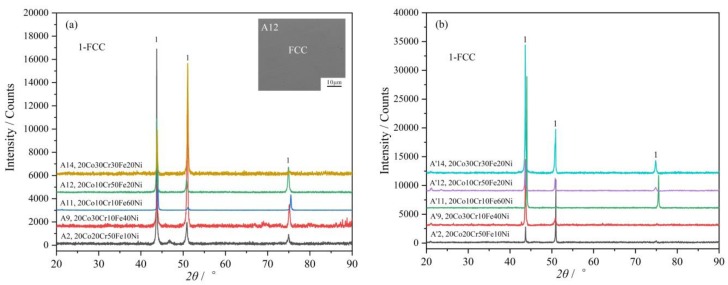
XRD patterns of some 20Co-Cr-Fe-Ni alloys which have single FCC phase in (**a**) as-cast state and (**b**) after being annealed at 1000 °C for 30 days. The inset figure is the BSE micrograph of the alloy A12.

As clearly shown in Figure 1, the 1000 °C isothermal section of the Co-Cr-Fe-Ni system with fixed 20 at.% Co has very large FCC single-phase region. There are seven designed alloys composed of a single FCC phase, i.e., A2 and A8–A13. Some selected XRD patterns of the as-casted alloys and 1000 °C annealed alloys are shown in Figure 2a,b, respectively. It needs to point out that the phase composition is uniform in these as-cast alloys. Take alloy A12 as an example, its Back Scattered Electron (BSE) micrograph is shown in the inset figure of Figure 2a. 

**Figure 3:**
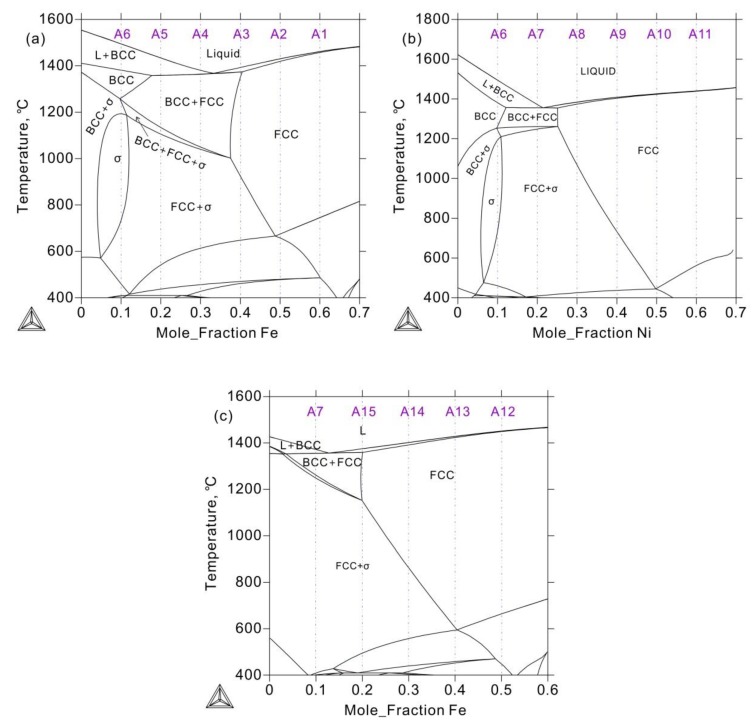
The vertical sections of the Co-Cr-Fe-Ni system calculated with the TCFE thermodynamic database. (**a**) fixed 20 at.%Co and 10 at.%Ni, (**b**) fixed 20 at.%Co and 10 at.%Fe, (**c**) fixed 20 at.%Co and 20 at.%Ni.

To clearly understand this phenomenon, the vertical section of the Co_2_Cr*_y_*Fe_6−*y*_Ni_2_ and Co_2_Cr_z_FeNi_7-z_ systems were thermodynamically calculated and presented in Figure 3. Although the FCC phase is formed by homogenization precipitation during solidification, the solidus is closed to the liquidus above the FCC phase region. The cooling rate of the sample in an arc-melting furnace is fast enough to ignore this temperature difference. Therefore, the alloy is solidified at the same time which leads to uniform composition. For the FCC phase is thermodynamically stable, there no phase precipitation or composition changing even annealed at 1000 °C for 30 days. Moreover, as can be seen from Figure 2, the full width at half maxima (FWHM) of the FCC phase after annealing becomes much narrower, which indicates that the internal stress decreased.

### 3.2. The Other Alloys Without Single FCC Phase

**Figure 4:**
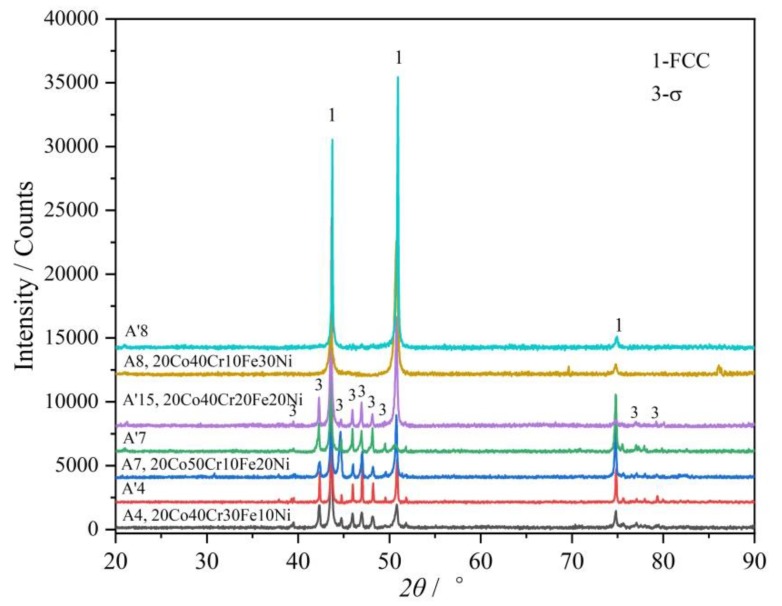
XRD patterns of the alloys A4, A7 and A8 in as-cast and 1000 °C annealed states and the alloy A’15 in 1000 °C annealed state. FCC and σ were found in these alloys.

**Figure 5:**
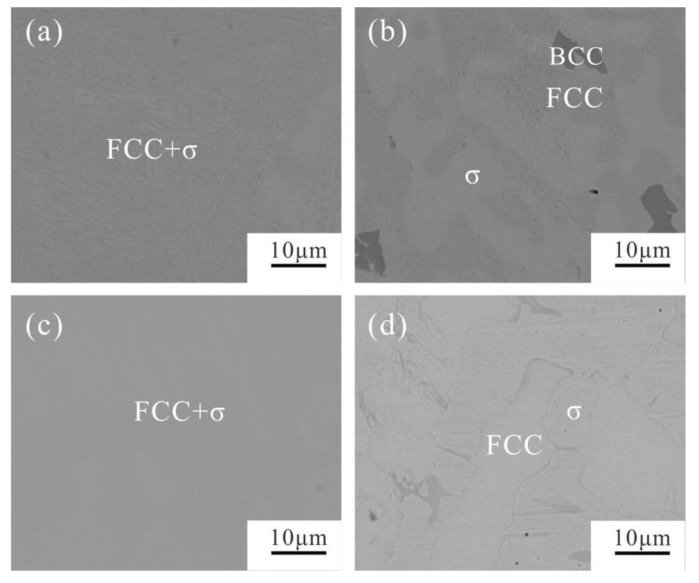
BSE micrographs of the alloys A4 (20Co40Cr30Fe10Ni) and A7 (20Co50Cr10Fe20Ni). (**a**) as-cast alloy A4, (**b**) 1000 annealed alloy A’4, (**c**) as-cast alloy A7, (**d**) 1000 annealed alloy A’7.

The alloys A4 (20Co40Cr30Fe10Ni) and A7 (20Co50Cr10Fe20Ni), whether in as-cast state or in 1000 °C annealed state, are composed of the FCC and σ phases. Their XRD patterns and BSE micrographs are shown in Figure 4 and Figure 5, respectively. As clearly shown in Figure 4, the FCC and σ phases can be well indexed in their XRD patterns. It indicates that the volume fraction of the σ phase is large enough. This agrees well with the calculated 1000 °C isothermal section in Figure 1. 

However, the microstructure of the alloys A4 (20Co40Cr30Fe10Ni) and A7 (20Co50Cr10Fe20Ni) change dramatically after being annealed at 1000 °C for 30 days, as shown in Figure 5. The color difference between these two phases is too limited to be well identified by SEM-EDS. After being annealed at 1000 °C for 30 days, the phase boundary becomes much clearer (Figure 5b,d). The detected phase compositions are presented in Table 1. It is clear that the Cr content of the σ phase in the alloy A’4 is 50.3 at.%, and in alloy A’7 reaches 61.2 at.%, which agrees well with the calculated 1000 °C isothermal section (dotted line in Figure 1). The BCC phase is a Cr-rich one with 87.7 at.% Cr. Compared to the as-cast microstructure, Cr atoms will precipitate from the FCC substrate and gather together to form the BCC phase after annealing at 1000 °C for 30 days. However, its volume fraction is too limited to be indexed in the XRD pattern.

As for the alloy A8 (20Co40Cr10Fe30Ni), the as-cast alloy A8 shows a single FCC phase. After being annealed at 1000 °C for 30 days, a small amount of the characteristic peaks of the σ phase were observed, as shown in Figure 4. However, the volume fraction of the σ phase is too limited to be well indexed. Moreover, it also cannot be well identified by SEM-EDS. According to the calculated 1000 °C section in Figure 1, because alloy A8 is near the FCC phase region boundary, it should just have a small amount of σ phase. 

**Figure 6:**
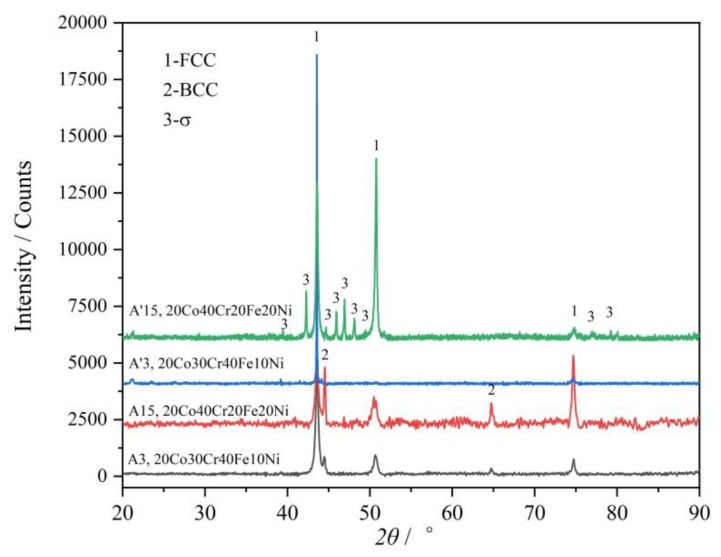
XRD patterns of the alloys A3 and A15 before and after annealing 1000 °C for 30 days.

**Figure 7:**
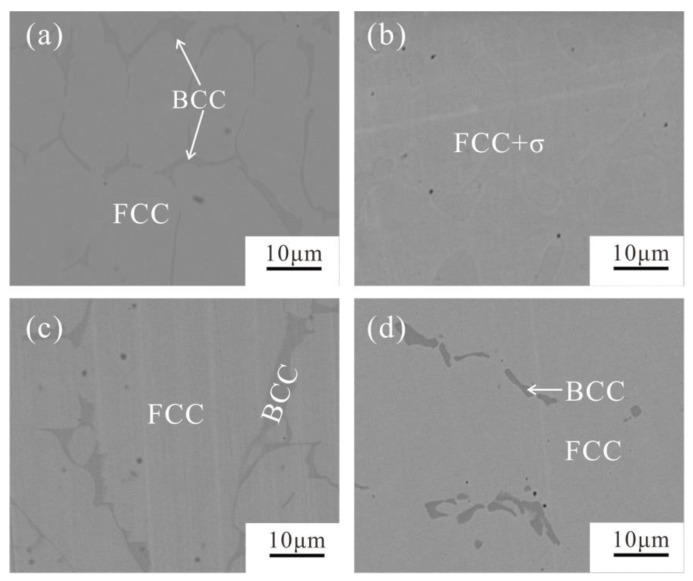
BSE micrographs of the alloys A3 and A15. (**a**) as-cast alloy A3, (**b**) 1000 annealed alloy A’3, (**c**) as-cast alloy A15, (**d**) 1000 annealed alloy A’15.

Similar to alloy A8, alloy A15 (20Co40Cr20Fe20Ni) also near the FCC phase region boundary. The BCC phase was indexed in the as-cast alloy A15, as shown in Figure 6. As clearly shown in Figure 7c, the net like BCC phase may exist in the grain boundary of the FCC phase. The detected composition of the BCC phase contains 55.0 at.% Cr, which is near the composition of the σ phase. However, after annealing at 1000 °C for 30 days, the BCC phase disappeared and a small amount of the σ phase could be detected, as shown in Figure 7d. The characteristic peaks of the σ phase can be clearly identified in its XRD (Figure 6).

Both the XRD and the BSE results proved that the FCC and a small amount of the BCC phase exist in the alloy A’3 (20Co30Cr40Fe10Ni), no matter the as-cast alloy or the annealed alloy, as shown in Figure 6 and Figure 7. However, after annealing at 1000 °C for 30 days, the Cr content in the BCC phase reaches 81.6 at.%, while the other elements decrease, as shown in Table 2, and the volume fraction of the BCC phase decreases. This means the as-cast BCC phase is supersaturated with Co, Fe, and Ni. 

**Figure 8:**
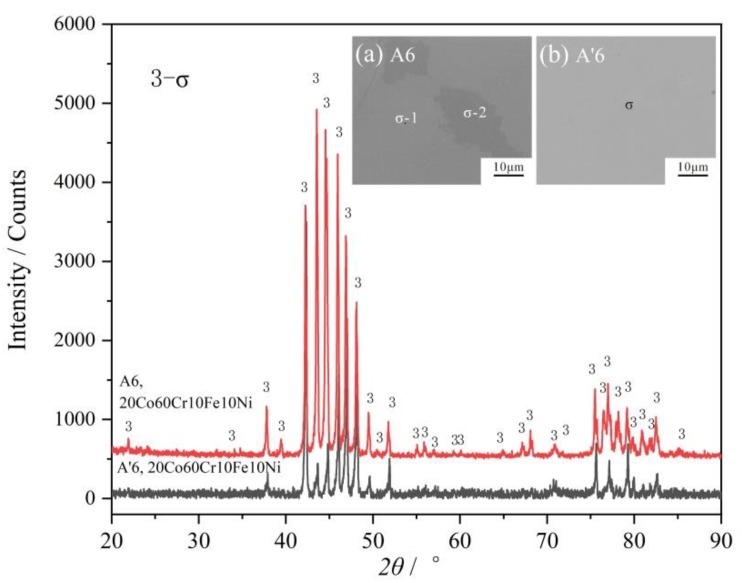
XRD patterns of the alloys A6 before and after annealing 1000 °C for 30 days. The inset figures are the related BSE micrographs.

Although two regions with a color difference exists in the BSE micrograph of the as-cast alloy A6, its XRD patterns just show a single σ phase. Their chemical compositions are listed in Table 2. The darker region has higher Cr content and lower Fe and Ni content. Therefore, σ-1 and σ-2 are noted in Figure 8. This phenomenon is caused by a large temperature range between the solidus and liquidus, which is different from that above the FCC phase region. After being annealed at 1000 °C for 30 days, the phase composition becomes uniform, as shown in Figure 8. For there is just one single σ phase, the alloy A6 (20Co60Cr10Fe10Ni) is much brittle. Of course, it cannot be used as a structure material.

As presented above, the σ phase is detected in the as-cast alloys A4–8 and A15. However, according to the calculated vertical sections in Figure 4, the BCC + FCC or FCC phase would exist in these as-cast alloys. That is to say, although thermodynamic calculation based on the TCFE database can predict the 1000 °C isothermal section, it fails to predict the stability of the σ phase at high temperature.

**Figure 9:**
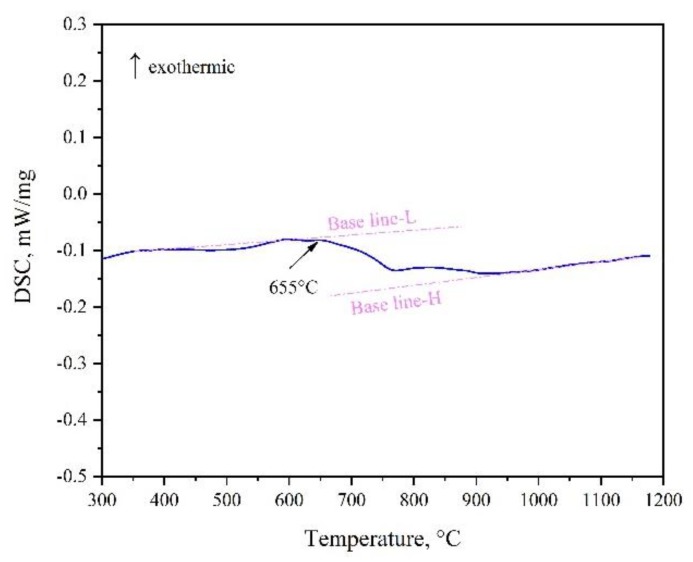
DSC curve of alloy A1 with a heating rate of 10 °C/min.

Moreover, XRD patterns of the as-cast and 1000 °C annealed Fe-rich alloy A1 (20Co10Cr60Fe10Ni) indicated that there were composed of the BCC and FCC phases. However, there is no color or composition difference between them. It seems conflict to the calculated 1000 °C isothermal section. It also needs to be pointed out that the examined alloys were cooled to room temperature. According to Figure 4b, the FCC phase in alloy A1 just stable above 700 °C, it will transform to the BCC phase below this temperature. DSC (Differential Scanning Calorimeter) curve in Figure 9 confirmed this transformation. During heating, BCC in the alloy transforms to FCC at 655–700 °C. Therefore, the detected phase in alloy A1 is different from the calculated isothermal section in Figure 1. In the Fe-rich alloys, we should carefully identify this transformation. As shown in Figure 1, the phase constituent of the 1000 °C annealed alloys agree well with the calculated isothermal section based on the TCFE database, especially for the FCC phase region. That is to say, the transformation of the FCC phase in the Co-Cr-Fe-Ni system can be predicted by the TCFE database.

### 3.3. Micro-Hardness

**Figure 10:**
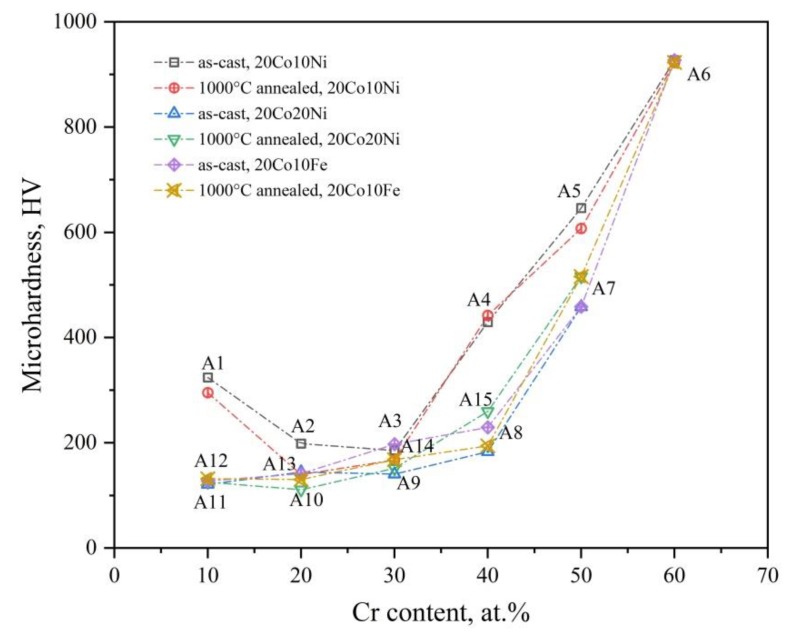
The micro-hardness of the 20Co-Cr-Fe-Ni alloys in as-cast and 1000 °C annealed states. The Fe-rich alloy A1 transforms from FCC to BCC during cooling and leads to higher microhardness.

Figure 10 shows the micro-hardness of the investigated 20Co-Cr-Fe-Ni alloys in the present work. There are three serials alloys, Co_2_Cr_x_Fe_7-x_Ni, Co_2_Cr_y_Fe_6-y_Ni_2_, Co_2_Cr*_z_*FeNi_7−*z*_. The Cr content of these serials alloys is set as the x axis. Except for the Fe-rich alloy A1 (20Co10Cr60Fe10Ni), which is a single BCC phase alloy with 310 HV, the microhardness of the other alloys increase with the Cr content when the Co and Ni or the Co and Fe contents are fixed. When the Cr content is below 30 at.%, the alloys are a single FCC phase, and the hardness of alloys changes slightly from 127 HV (alloy A11, 20Co10Cr10Fe60Ni) to 197 HV (alloys A9, 20Co30Cr10Fe40Ni, A14, 20Co30Cr30Fe20Ni). However, the hardness suddenly increases when the Cr content is more than 40 at.%. It is because the volume fraction of the σ phase increases with the Cr content in these three serials of alloys, which can dramatically harden the alloys. However, the alloy is very brittle when it is a single σ phase (alloy A6, 20Co60Cr10Fe10Ni, 927 HV). Obviously, the hard effect of the BCC phase in these FCC based alloys is much lower than the σ phase.

Annealing at 1000 °C for 30 days has limited effect on the hardness of the 20Co-Cr-Fe-Ni alloys, especially for the alloys have a single phase. As discussed above, a uniform FCC phase can be obtained in alloy A9–A14, and there is no phase composition change after annealing. Therefore, the alloy property is the same as the as-cast one. However, as for the alloys with two or three phases, annealing at 1000 °C for 30 days can slightly decreases the microhardness of the alloys. It is because of the decreased volume fraction of the σ phase and the coarsened grains.

## 4. Conclusions

The phase constituent and microhardness of the 20Co-Cr-Fe-Ni alloys under the as-cast and annealed at 1000 °C for 30 days states were investigated in the present work. The following conclusions can be drawn:

(1) When the Cr content is less than 30 at.%, a uniform stable single FCC phase can be obtained in as-cast 20Co-Cr-Fe-Ni (<60 at.%Fe) alloys. Annealing at 1000 °C has no effect on their phase composition and microhardness.
(2) The σ phase forms when the Cr content is above 40 at.% in the 20Co-Cr-Fe-Ni alloys. Its volume fraction increased with the Cr content and leads to an increase of the microhardness.
(3) Annealing at 1000 °C for 30 days can decrease the volume fraction of the σ phase and coarsen the crystal grain.
(4) Except for the Fe-rich alloy A1 (20Co10Cr60Fe10Ni), the microhardness of the other alloys increases with the Cr content when the Co and Ni or the Co and Fe contents were fixed.
(5) Thermodynamic calculation based on the TCFE database can well predict the phase stability of the FCC phase and the 1000 °C isothermal section. However, it fails to predict the phase stability near the liquidus.

## Figures and Tables

**Table 1:** The detected chemical composition of the alloys A’4 (20Co40Cr30Fe10Ni) and A’7 (20Co50Cr10Fe20Ni) after being annealed at 1000 °C for 30 days.

Designed Composition	No.	Phases	Detected Composition (at.%)			
			Co	Cr	Fe	Ni
20Co40Cr30Fe10Ni	A’4	FCC	20.1	39.3	30.9	9.7
		BCC	3.5	87.7	6.8	2.0
		σ	17.5	50.3	25.9	6.3
20Co50Cr10Fe20Ni	A’7	FCC	21.9	44.1	10.8	23.2
		σ	16.7	61.2	8.9	13.2

**Table 2:** The detected chemical composition of the alloys A3, A6, and A15 in both as-cast and 1000 °C annealed states.

Designed Composition	No.	State	Phases	Detected Composition (at.%)			
				Co	Cr	Fe	Ni
20Co30Cr40Fe10Ni	A3	as-cast	total	22.5	29.9	38.9	8.7
			FCC	20.0	28.6	41.5	9.9
			BCC	14.7	42.0	38.1	5.2
	A’3	1000 °C annealed	FCC	20.2	29.4	39.7	10.7
			BCC	4.3	81.6	11.6	2.5
20Co40Cr20Fe20Ni	A15	as-cast	total	20.1	40	19.7	20.2
			FCC	20.4	38.6	20.5	20.5
			BCC	15.5	55.0	18.2	11.3
	A’15	1000 °C annealed	FCC	20.6	36.0	20.8	22.6
			σ	17.1	55.3	16.3	11.3
20Co60Cr10Fe10Ni	A6	as-cast	total	19.9	59.9	9.8	10.4
			σ-1	23.1	54.3	10.4	12.2
			σ-2	17.9	65.2	10.1	6.8
	A’6	1000 °C annealed	σ	20.0	59.7	9.8	10.5
